# 2-(6-Chloro-1*H*-indol-3-yl)acetonitrile

**DOI:** 10.1107/S1600536811053372

**Published:** 2011-12-17

**Authors:** Jin-Feng Li, Yang-Hui Luo

**Affiliations:** a Key Laboratory of Urban and Architectural Heritage Conservation, (Southeast University), Ministry of Education, Nanjing 210096, People’s Republic of China, and, College of Chemistry and Chemical Engineering, Southeast University, Nanjing 210096, People’s Republic of China

## Abstract

In the title compound, C_10_H_7_ClN_2_, the carbonitrile group is twisted away from the plane of the indole ring system [C_cy_—C_me_—C_ar_—C_ar_ = −44.7 (8)°; cy = cyanide, me = methyl­ene and ar = aromatic]. In the crystal, N—H⋯N hydrogen bonds link the mol­ecules into *C*(7) chains propagating in [010]. Aromatic π–π stacking inter­actions [minimum centroid–centroid separation = 3.663 (3) Å] are also observed.

## Related literature

For a related structure, see: Ge *et al.* (2012 ▶).
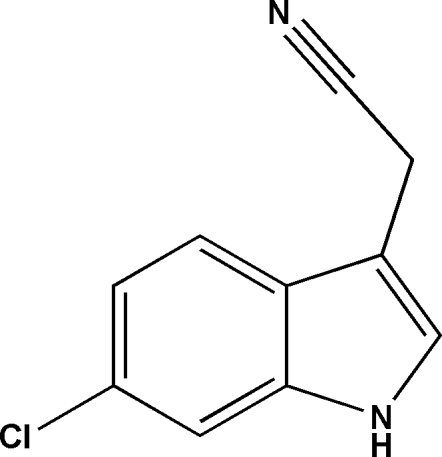


## Experimental

### 

#### Crystal data

C_10_H_7_ClN_2_
*M* *_r_* = 190.63
Monoclinic, 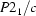

*a* = 9.761 (2) Å
*b* = 11.205 (2) Å
*c* = 8.7791 (18) Å
β = 100.39 (3)°
*V* = 944.4 (3) Å^3^
*Z* = 4
Mo *K*α radiation
μ = 0.35 mm^−1^
*T* = 293 K
0.20 × 0.12 × 0.10 mm

#### Data collection

Rigaku SCXmini CCD diffractometer
Absorption correction: multi-scan (*CrystalClear*; Rigaku, 2005 ▶) *T*_min_ = 0.950, *T*_max_ = 0.965
8938 measured reflections
2122 independent reflections
1247 reflections with *I* > 2σ(*I*)
*R*_int_ = 0.134

#### Refinement

*R*[*F* ^2^ > 2σ(*F* ^2^)] = 0.110
*wR*(*F* ^2^) = 0.312
*S* = 1.05
2122 reflections
118 parameters
H-atom parameters constrained
Δρ_max_ = 0.51 e Å^−3^
Δρ_min_ = −0.46 e Å^−3^

### 

Data collection: *CrystalClear* (Rigaku, 2005 ▶); cell refinement: *CrystalClear*; data reduction: *CrystalClear*; program(s) used to solve structure: *SHELXS97* (Sheldrick, 2008 ▶); program(s) used to refine structure: *SHELXL97* (Sheldrick, 2008 ▶); molecular graphics: *DIAMOND* (Brandenburg & Putz, 2005 ▶); software used to prepare material for publication: *SHELXL97*.

## Supplementary Material

Crystal structure: contains datablock(s) I, global. DOI: 10.1107/S1600536811053372/hb6557sup1.cif

Structure factors: contains datablock(s) I. DOI: 10.1107/S1600536811053372/hb6557Isup2.hkl

Supplementary material file. DOI: 10.1107/S1600536811053372/hb6557Isup3.cml

Additional supplementary materials:  crystallographic information; 3D view; checkCIF report

## Figures and Tables

**Table 1:** Hydrogen-bond geometry (Å, °)

*D*—H⋯*A*	*D*—H	H⋯*A*	*D*⋯*A*	*D*—H⋯*A*
N2—H4*A*⋯N1^i^	0.86	2.23	3.016 (6)	151

Symmetry code: (i) 
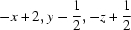
.

**Fig. 1.**
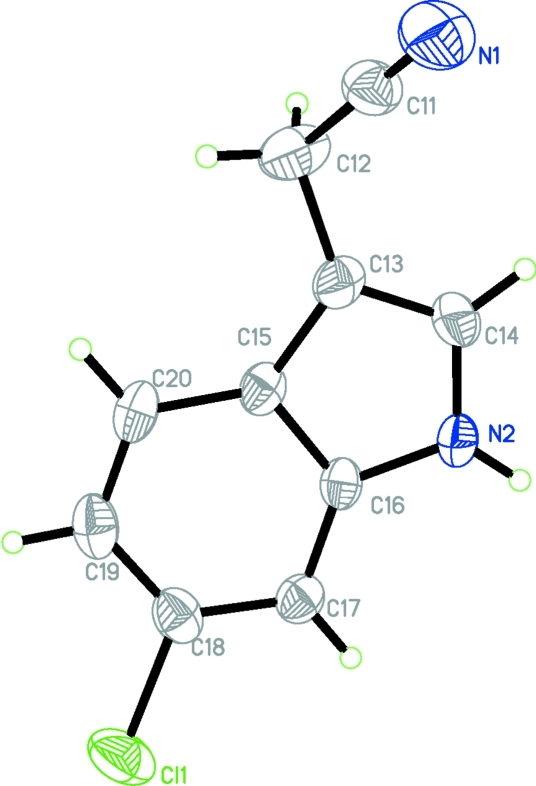
The molecular structure of the title compound with displacement ellipsoids drawn at the 30% probability level.

**Fig. 2.**
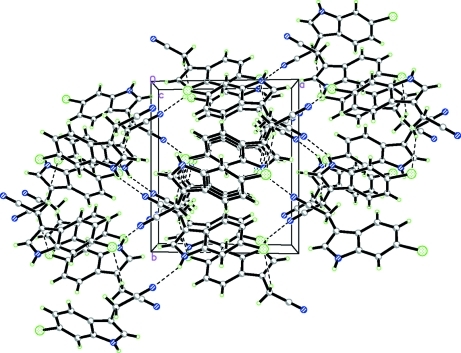
A packing view down the a axis showing hydrogen bonds as dashed lines.

## supplementary crystallographic information

### Experimental

The title compound was obtained commercially from ChemFuture PharmaTech, Ltd (Nanjing, Jiangsu). and were used as received without further purification. Colourless prisms were obtained by slow evaporation of a methanol solution.

### Refinement

All H atoms attached to C atoms and N atoms were fixed geometrically and treated as riding with C—H = 0.93 Å (CH), C—H = 0.97 Å (CH_2_) and N—H = 0.86 Å with *U*_iso_(H) =1.2*U*_eq_.

### Crystal data

**Table:**

C_10_H_7_ClN_2_	*F*(000) = 392
*M**_r_* = 190.63	*D*_x_ = 1.341 Mg m^−^^3^
Monoclinic, *P*2_1_/*c*	Mo *K*α radiation, λ = 0.71073 Å
Hall symbol: -P 2ybc	Cell parameters from 2122 reflections
*a* = 9.761 (2) Å	θ = 3.0–27.5°
*b* = 11.205 (2) Å	µ = 0.35 mm^−^^1^
*c* = 8.7791 (18) Å	*T* = 293 K
β = 100.39 (3)°	Prism, colourless
*V* = 944.4 (3) Å^3^	0.20 × 0.12 × 0.10 mm
*Z* = 4	

### Data collection

**Table:**

Rigaku SCXmini CCD diffractometer	2122 independent reflections
Radiation source: fine-focus sealed tube	1247 reflections with *I* > 2σ(*I*)
graphite	*R*_int_ = 0.134
Detector resolution: 13.6612 pixels mm^-1^	θ_max_ = 27.5°, θ_min_ = 3.0°
CCD_Profile_fitting scans	*h* = −12→12
Absorption correction: multi-scan (*CrystalClear*; Rigaku, 2005)	*k* = −14→13
*T*_min_ = 0.950, *T*_max_ = 0.965	*l* = −11→11
8938 measured reflections	

### Refinement

**Table:**

Refinement on *F*^2^	Primary atom site location: structure-invariant direct methods
Least-squares matrix: full	Secondary atom site location: difference Fourier map
*R*[*F*^2^ > 2σ(*F*^2^)] = 0.110	Hydrogen site location: inferred from neighbouring sites
*wR*(*F*^2^) = 0.312	H-atom parameters constrained
*S* = 1.05	*w* = 1/[σ^2^(*F*_o_^2^) + (0.1465*P*)^2^ + 0.5597*P*] where *P* = (*F*_o_^2^ + 2*F*_c_^2^)/3
2122 reflections	(Δ/σ)_max_ < 0.001
118 parameters	Δρ_max_ = 0.51 e Å^−^^3^
0 restraints	Δρ_min_ = −0.46 e Å^−^^3^

### Special details

**Table:**

Geometry. All e.s.d.'s (except the e.s.d. in the dihedral angle between two l.s. planes) are estimated using the full covariance matrix. The cell e.s.d.'s are taken into account individually in the estimation of e.s.d.'s in distances, angles and torsion angles; correlations between e.s.d.'s in cell parameters are only used when they are defined by crystal symmetry. An approximate (isotropic) treatment of cell e.s.d.'s is used for estimating e.s.d.'s involving l.s. planes.
Refinement. Refinement of *F*^2^ against ALL reflections. The weighted *R*-factor *wR* and goodness of fit *S* are based on *F*^2^, conventional *R*-factors *R* are based on *F*, with *F* set to zero for negative *F*^2^. The threshold expression of *F*^2^ > σ(*F*^2^) is used only for calculating *R*-factors(gt) *etc*. and is not relevant to the choice of reflections for refinement. *R*-factors based on *F*^2^ are statistically about twice as large as those based on *F*, and *R*- factors based on ALL data will be even larger.

### Fractional atomic coordinates and isotropic or equivalent isotropic displacement parameters (Å^2^)

**Table:**

	*x*	*y*	*z*	*U*_iso_*/*U*_eq_	
Cl1	0.24329 (14)	0.04179 (19)	0.32068 (19)	0.0959 (8)	
N1	1.0357 (5)	0.3256 (5)	0.0579 (6)	0.0929 (16)	
N2	0.7608 (4)	−0.0082 (4)	0.2478 (4)	0.0578 (10)	
H4A	0.7910	−0.0635	0.3133	0.069*	
C11	0.9347 (5)	0.2792 (4)	0.0093 (5)	0.0628 (12)	
C12	0.8029 (6)	0.2208 (6)	−0.0508 (6)	0.0842 (18)	
H12A	0.8115	0.1777	−0.1445	0.101*	
H12B	0.7314	0.2810	−0.0781	0.101*	
C13	0.7581 (4)	0.1343 (4)	0.0646 (5)	0.0578 (12)	
C14	0.8382 (5)	0.0517 (5)	0.1549 (6)	0.0673 (14)	
H14A	0.9319	0.0378	0.1539	0.081*	
C15	0.6203 (4)	0.1262 (4)	0.1028 (4)	0.0481 (10)	
C16	0.6263 (4)	0.0360 (3)	0.2172 (5)	0.0467 (10)	
C17	0.5117 (4)	0.0061 (4)	0.2868 (5)	0.0519 (11)	
H17A	0.5167	−0.0525	0.3627	0.062*	
C18	0.3907 (5)	0.0705 (4)	0.2342 (5)	0.0588 (12)	
C19	0.3788 (5)	0.1573 (4)	0.1185 (6)	0.0646 (13)	
H19A	0.2943	0.1961	0.0862	0.078*	
C20	0.4923 (5)	0.1854 (4)	0.0522 (5)	0.0568 (11)	
H20A	0.4848	0.2430	−0.0252	0.068*	

### Atomic displacement parameters (Å^2^)

**Table:**

	*U*^11^	*U*^22^	*U*^33^	*U*^12^	*U*^13^	*U*^23^
Cl1	0.0512 (9)	0.1570 (18)	0.0826 (11)	−0.0064 (8)	0.0201 (7)	−0.0179 (9)
N1	0.070 (3)	0.108 (4)	0.096 (4)	−0.037 (3)	0.003 (2)	0.003 (3)
N2	0.042 (2)	0.073 (2)	0.054 (2)	0.0056 (18)	−0.0036 (15)	0.0129 (18)
C11	0.056 (3)	0.067 (3)	0.067 (3)	−0.009 (2)	0.013 (2)	0.001 (2)
C12	0.078 (3)	0.111 (4)	0.056 (3)	−0.046 (3)	−0.006 (2)	0.014 (3)
C13	0.050 (2)	0.074 (3)	0.044 (2)	−0.018 (2)	−0.0083 (17)	0.0047 (19)
C14	0.038 (2)	0.097 (4)	0.064 (3)	−0.003 (2)	0.001 (2)	0.005 (2)
C15	0.046 (2)	0.051 (2)	0.042 (2)	−0.0085 (18)	−0.0082 (16)	−0.0029 (16)
C16	0.041 (2)	0.049 (2)	0.046 (2)	−0.0003 (17)	−0.0047 (16)	−0.0014 (16)
C17	0.054 (3)	0.054 (2)	0.046 (2)	−0.003 (2)	0.0033 (19)	0.0004 (18)
C18	0.047 (2)	0.067 (3)	0.061 (3)	−0.001 (2)	0.0034 (19)	−0.013 (2)
C19	0.053 (3)	0.066 (3)	0.068 (3)	0.013 (2)	−0.008 (2)	−0.012 (2)
C20	0.062 (3)	0.047 (2)	0.053 (2)	0.003 (2)	−0.0123 (19)	0.0017 (17)

### Geometric parameters (Å, °)

**Table:**

Cl1—C18	1.773 (5)	C14—H14A	0.9300
N1—C11	1.128 (6)	C15—C20	1.413 (6)
N2—C14	1.381 (6)	C15—C16	1.419 (6)
N2—C16	1.384 (5)	C16—C17	1.408 (6)
N2—H4A	0.8600	C17—C18	1.390 (6)
C11—C12	1.455 (7)	C17—H17A	0.9300
C12—C13	1.522 (6)	C18—C19	1.396 (7)
C12—H12A	0.9700	C19—C20	1.379 (7)
C12—H12B	0.9700	C19—H19A	0.9300
C13—C14	1.368 (7)	C20—H20A	0.9300
C13—C15	1.447 (6)		
			
C14—N2—C16	108.2 (4)	C20—C15—C13	134.7 (4)
C14—N2—H4A	125.9	C16—C15—C13	106.7 (4)
C16—N2—H4A	125.9	N2—C16—C17	129.1 (4)
N1—C11—C12	178.7 (6)	N2—C16—C15	108.0 (4)
C11—C12—C13	112.7 (4)	C17—C16—C15	122.8 (4)
C11—C12—H12A	109.0	C18—C17—C16	115.2 (4)
C13—C12—H12A	109.0	C18—C17—H17A	122.4
C11—C12—H12B	109.0	C16—C17—H17A	122.4
C13—C12—H12B	109.0	C17—C18—C19	123.9 (4)
H12A—C12—H12B	107.8	C17—C18—Cl1	118.0 (4)
C14—C13—C15	106.3 (4)	C19—C18—Cl1	118.0 (4)
C14—C13—C12	127.8 (5)	C20—C19—C18	119.9 (4)
C15—C13—C12	125.8 (4)	C20—C19—H19A	120.0
C13—C14—N2	110.8 (4)	C18—C19—H19A	120.0
C13—C14—H14A	124.6	C19—C20—C15	119.5 (4)
N2—C14—H14A	124.6	C19—C20—H20A	120.3
C20—C15—C16	118.6 (4)	C15—C20—H20A	120.3
			
N1—C11—C12—C13	−68 (28)	C13—C15—C16—N2	0.3 (4)
C11—C12—C13—C14	−44.7 (8)	C20—C15—C16—C17	−2.5 (6)
C11—C12—C13—C15	133.6 (5)	C13—C15—C16—C17	177.6 (4)
C15—C13—C14—N2	0.0 (6)	N2—C16—C17—C18	177.5 (4)
C12—C13—C14—N2	178.6 (4)	C15—C16—C17—C18	0.8 (6)
C16—N2—C14—C13	0.2 (6)	C16—C17—C18—C19	1.3 (6)
C14—C13—C15—C20	−180.0 (5)	C16—C17—C18—Cl1	−177.9 (3)
C12—C13—C15—C20	1.4 (8)	C17—C18—C19—C20	−1.6 (7)
C14—C13—C15—C16	−0.1 (5)	Cl1—C18—C19—C20	177.6 (4)
C12—C13—C15—C16	−178.8 (4)	C18—C19—C20—C15	−0.3 (7)
C14—N2—C16—C17	−177.4 (4)	C16—C15—C20—C19	2.2 (6)
C14—N2—C16—C15	−0.3 (5)	C13—C15—C20—C19	−178.0 (5)
C20—C15—C16—N2	−179.8 (4)		

### Hydrogen-bond geometry (Å, °)

**Table:**

*D*—H···*A*	*D*—H	H···*A*	*D*···*A*	*D*—H···*A*
N2—H4A···N1^i^	0.86	2.23	3.016 (6)	151

Symmetry codes: (i) −*x*+2, *y*−1/2, −*z*+1/2.
